# Can Emergency Physicians Perform Common Carotid Doppler Flow Measurements to Assess Volume Responsiveness?

**DOI:** 10.5811/westjem.2015.1.24301

**Published:** 2015-02-26

**Authors:** Lori A. Stolz, Jarrod M. Mosier, Austin M. Gross, Matthew J. Douglas, Michael Blaivas, Srikar Adhikari

**Affiliations:** * University of Arizona, Department of Emergency Medicine, Tucson, Arizona; † University of Arizona, Department of Emergency Medicine and Internal Medicine, Tucson, Arizona; ‡ Evergreen Emergency Services, Department of Emergency Medicine, Kirkland, Washington; § St. Francis Hospital, Department of Emergency Medicine, Columbus, Georgia

## Abstract

**Introduction:**

Common carotid flow measurements may be clinically useful to determine volume responsiveness. The objective of this study was to assess the ability of emergency physicians (EP) to obtain sonographic images and measurements of the common carotid artery velocity time integral (VTi) for potential use in assessing volume responsiveness in the clinical setting.

**Methods:**

In this prospective observational study, we showed a five-minute instructional video demonstrating a technique to obtain common carotid ultrasound images and measure the common carotid VTi to emergency medicine (EM) residents. Participants were then asked to image the common carotid artery and obtain VTi measurements. Expert sonographers observed participants imaging in real time and recorded their performance on nine performance measures. An expert sonographer graded image quality. Participants were timed and answered questions regarding ease of examination and their confidence in obtaining the images.

**Results:**

A total of 30 EM residents participated in this study and each performed the examination twice. Average time required to complete one examination was 2.9 minutes (95% CI [2.4–3.4 min]). Participants successfully completed all performance measures greater than 75% of the time, with the exception of obtaining measurements during systole, which was completed in 65% of examinations. Median resident overall confidence in accurately performing carotid VTi measurements was 3 (on a scale of 1 [not confident] to 5 [confident]).

**Conclusion:**

EM residents at our institution learned the technique for obtaining common carotid artery Doppler flow measurements after viewing a brief instructional video. When assessed at performing this examination, they completed several performance measures with greater than 75% success. No differences were found between novice and experienced groups.

## INTRODUCTION

The search for a reliable, accurate and non-invasive measure of volume responsiveness in patients in shock is an ongoing endeavor. Shock is a common presentation in the emergency department (ED) and emergency physicians (EP) are often confronted with the challenge of accurate volume assessment and adequate volume resuscitation. In septic patients in particular, improvements in mortality have been linked to early and aggressive volume resuscitation.^1^,^2^ However, recent research also demonstrates increased mortality and increased length of stay in patients with over-aggressive volume resuscitation.^3^,^4^ Therefore, it is important for EPs to have accurate diagnostic tools to determine which patient will have an increase in their stroke volume, and therefore cardiac output, with additional intravenous fluids. This concept is frequently termed volume responsiveness. In practice, a volume-responsive patient will have improved hemodynamics with fluid administration. Technically, a volume-responsive patient will have at least a 15% increase in stroke volume with a 500mL bolus of crystalloid fluids. Critically ill, unstable patients who are not volume responsive, but are given additional fluids, will not improve their hemodynamics and may, in fact, be harmed via the resulting volume overload.

Currently available methods of measuring volume responsiveness have limitations. Central venous pressure (CVP) has been the primary means of guiding volume resuscitation in sepsis since the inception of early goal-directed therapy. CVP measurements have been shown to be an inaccurate predictor of volume responsiveness in several studies and have low rates of physician use within sepsis bundles.^5^–^7^ Pulmonary artery occlusion pressure (wedge pressure) is invasive, can also be inaccurate and is rarely used in the ED.^8^ Likewise, sonographic inferior vena cava measurements may not accurately predict fluid responsiveness.^9^,^10^ Additionally, transthoracic echocardiography can be used to measure cardiac output and, in turn, volume responsiveness; however, it is highly operator dependent with poor test-re-test reliability.^11^

One recent study has shown that common carotid velocity time integral (VTi) measurements can be used with a passive leg raise maneuver to determine volume responsiveness in critically ill patients.^12^ The measurements are non-invasive and were found to be accurate in the prediction of volume responsiveness in comparison with a non-invasive cardiac output monitor. This technique has obvious appeal as a useful clinical tool in the ED and could improve upon the currently available methods in use. It is non-invasive, repeatable and the structures of interest are superficial in location, thus easy to image. Sonographic measurements in the cited study were obtained by a vascular certified echocardiographer; however, the average EP does not possess the training or skills of an expert sonographer. The ability to generalize the utility of this technique to EPs with standard point-of-care (POC) ultrasound experience requires further investigation. Prior studies have evaluated the ability of EPs to learn various sonographic techniques with brief training interventions with results indicating success and accuracy with these brief interventions.^13^–^15^ The objective of this study was to determine the ability of emergency medicine (EM) residents to obtain sonographic images and measurements of the common carotid artery VTi for potential use in assessing volume responsiveness in the clinical setting.

## METHODS

### Study Design and Setting

We conducted this prospective observational study at two affiliated academic medical centers with two EM residency programs and a combined EM/pediatrics residency program. The ED has an active emergency ultrasound training program. Hospital credentialing in POC ultrasound is available for EPs and is based on American College of Emergency Physicians ultrasound guidelines.^16^ This study was reviewed by local institutional review committee and approved.

### Study Population

Participants in the study were volunteer EM resident physicians from all years of training. All residents from the three affiliated residency programs were invited to participate. None of these residents had any prior experience with carotid ultrasound.

### Study Protocol

**Figure 1:**
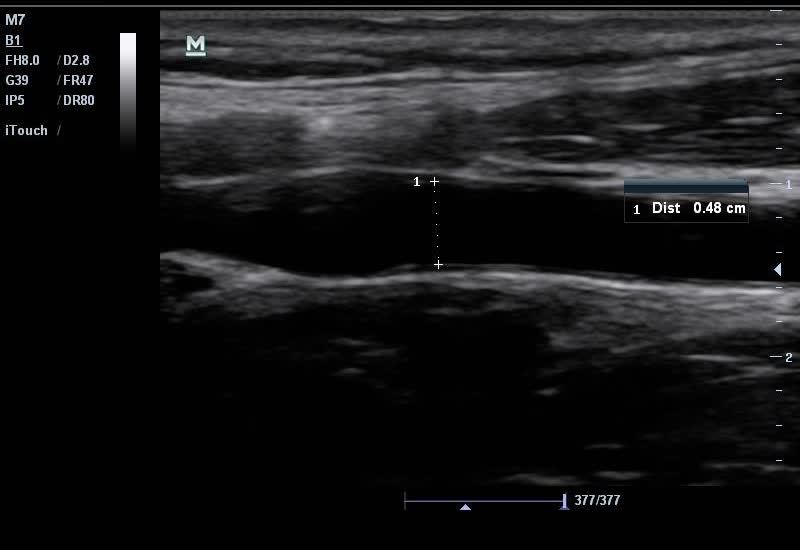
Ultrasound image of common carotid diameter measurement obtained within 0.5 cm of the carotid bulb and measured intima to intima.

**Figure 2:**
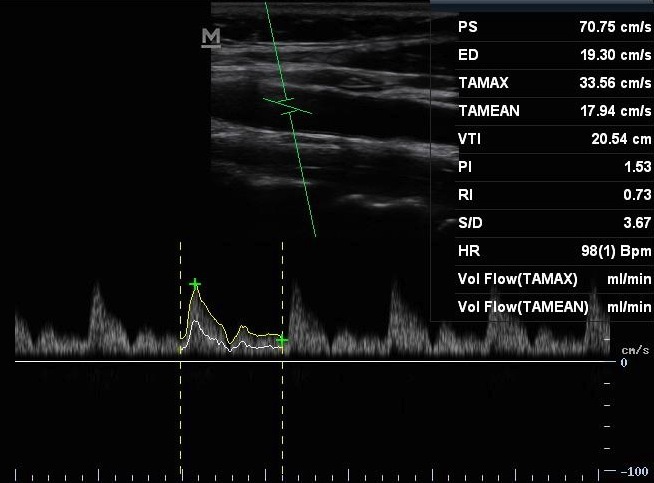
Ultrasound image of common carotid Doppler waveform and velocity time integral measurement acquired with an insonation angle of 58 degrees. The upper spectral waveform tracing (hollow arrow) represents peak flow velocity and the lower spectral waveform (solid arrow) represents mean flow velocity. Plus signs on the spectral waveform represent peak systolic velocity and end diastolic velocity.

We created a five-minute instructional video demonstrating a technique to obtain common carotid ultrasound images and measure the common carotid VTi based on previously published methods.^12^ The published technique involves obtaining antero-posterior measurements of the common carotid artery diameter in systole within approximately 0.5cm of the common carotid bulb in the long axis with a 12-7 MHz broadband linear array transducer (Figure 1, Video 1). The VTi is then determined through digitalized Doppler spectral envelopes with the sample obtained at the location that the diameter was taken. The Doppler gate is placed in the middle of the artery with a 45- to 60-degree angle of insonation (Figure 2, Video 2). We sent the instructional video by email to all potential participants to review, and it was available for them to review again prior to their performance of the examination. Images were obtained by each participant on two healthy volunteers using a Mindray M7 (Shenzhen, China). Two expert sonographers observed participants performing the technique in realtime. They recorded each participant’s performance on nine performance measures as a dichotomous variable, either as successfully completed or not. Participants filled out a questionnaire detailing the number of previous ultrasound examinations they had performed, their comfort level with the technique and their preference for the brief video as a learning method versus others. An additional expert sonographer who was blinded to the study hypothesis reviewed the images and assessed all images for Doppler sample volume placement, accuracy of measurement of common carotid artery diameter and image quality using a scale of 1 (poor image quality) - 5 (excellent image quality). All expert sonographers had performed greater than 1000 POC ultrasound examinations before the study period.

### Data Analysis

Data are presented as means, medians, or proportions as appropriate, along with 95% confidence intervals (CIs) or interquartile ranges. We used a paired t-test (time to examination completion) and McNemar’s test (performance measures) to test if outcomes differed between the two volunteer models. All confidence intervals for pooled data were calculated accounting for residents as clusters with two measurements (one for each model). No formal power calculation was conducted prior to the study. We used Stata v.12.1 (StataCorp, College Station, TX) for all analyses.

## RESULTS

A total of 30 EM residents participated in this study. The number of ultrasound examinations completed by residents prior to participation in the study was between 0–839 with a median of 23 (IQR: 11–154). Residents were stratified into experienced (n=9, 30%) and novice groups (n=21, 70%) based on greater or less than 125 ultrasound examinations previously performed. The proportion of participants in the study that were first-year residents was 80.0% (24/30), while the proportion of non-participants that were first-year residents was 20.2% (24/119; p<0.001). All of the participants watched the instructional video prior to performing the examination: 46% of participants watched it once, 46% watched it twice, and 8% watched it more than twice. The greatest number of times the video was reviewed was four.

There was no statistical difference for any outcome variable between the two volunteer models (p>0.1), so all data were pooled. Average time required for participants to complete one complete examination was 2.9 minutes (95% CI [2.4–3.4 min]). Mean time to examination completion was 3.0 minutes (95% CI [2.4–3.6 min]) for novices and 2.7 minutes (95% CI [2.0–3.4 min]) for more experienced residents with no significant difference in time to completion between groups.

**Figure 3:**
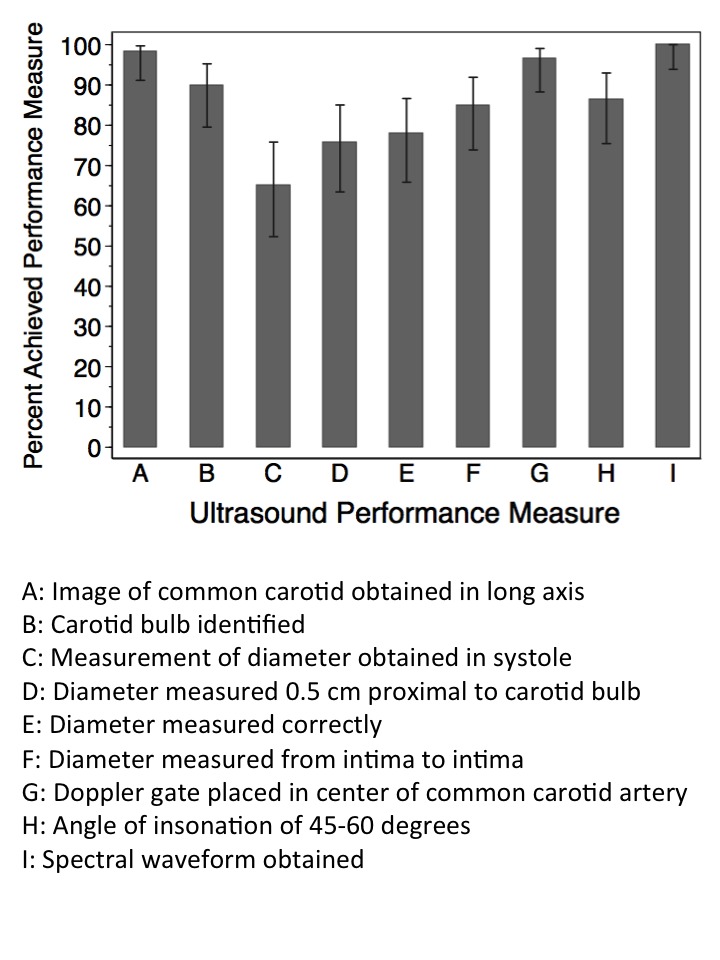
Percent successful completion of ultrasound performance measures. I-bars represent 95% confidence intervals. N = 30 subjects

The proportion of ultrasound examinations in which the physicians successfully completed each performance measure is shown in Figure 3. Residents were able to successfully complete all performance measures greater than 75% of the time, with the exception of obtaining carotid measurements during systole, which was only completed in 65% (95% CI [51.5–76.8%]) of examinations. We compared successful performance of each measure between novice and experienced groups. Obtaining an angle of insonation of 45- to 60-degrees was performed successfully more frequently by novice sonographers than experienced sonographers (92% versus 70%, p= 0.03). There was no difference between groups for other performance measures.

Median image quality, as reviewed by an expert sonologist, was 3 (IQR 3–4) on a scale of 1–5 for static carotid diameter measurements. Median image quality for carotid VTi spectral tracings was also 3 (IQR 2–4). There was no difference in image quality between the novice and experienced groups (p=0.98).

Median resident overall confidence in accurately performing carotid VTi measurements after this educational intervention (as rated on a scale of 1 [not confident] to 5 [confident]) was 3 (IQR: 3–4). Resident opinion of how technically challenging this technique was rated as a median of 2 (IQR: 2–3) on a scale of 1–5 (1=not challenging, 5=very challenging). All participants responded that it is feasible to obtain carotid Doppler flow measurements in the ED. Ninety-seven percent of residents answered that they would like to learn additional ultrasound techniques in this instructional video format.

## DISCUSSION

Accurate, non-invasive measurement of fluid responsiveness is elusive and yet critical for EPs; however, currently available methods have known shortcomings. Making accurate and timely decisions about volume resuscitation and vasopressor initiation is critical in numerous ED patients and in all categories of shock as both under-resuscitation and over-resuscitation are detrimental. Prior research by Marik et al.^12^ examined carotid flow velocities as a possible means of determining volume responsiveness. This technique could potentially improve EP assessment of volume responsiveness and is attractive because ultrasound is non-invasive, readily available in most acute care departments and performed at the bedside.

This study assessed the ability of a group of EM residents at our institution to perform carotid ultrasonographic examination and obtain Doppler measurements of carotid flow. EPs with minimal ultrasound experience learned this technique after a brief instructional video. After this training, they were able to complete each step of the examination greater than 75% of the time with the exception of measuring the carotid diameter during systole. It remains to be seen if residents can truly master this technique; however, there is undoubtedly a learning curve associated with mastery of this skill and improvement would be seen over time.

This is a new technique requiring accurate Doppler measurements, which some may consider an advanced skill. In contrast to our study, Marik et al.^12^ used one experienced echocardiographer to obtain all carotid flow measurements. The participants in our study were largely first-year residents and inexperienced sonographers. Even those with minimal ultrasound experience completed each performance measure greater than 75% for all performance measures except one. Among the physicians who learned and obtained the measurements in this study, there was no difference between experienced and novice sonographers suggesting that this technique does not require advanced skills. The ease in obtaining measurements is likely aided by the superficial location of the common carotid artery, the ease of identification of the structures and the ability to visualize the structures regardless of body habitus. These advantages may make this technique potentially preferable for monitoring volume status and responsiveness over other currently available techniques.

Very little time was required to obtain measurements of common carotid VTi, making this an attractive alternative to existing techniques. Time is of the essence when making a decision to initiate vasopressors on a critically ill patient, and the rapid pace of a typical ED demands quick decision-making and assessment. Other techniques take more time, are dependent on patient positioning, body habitus and present challenges.

This study highlights key areas that may require additional emphasis while teaching this technique in the future, namely obtaining the common carotid diameter in systole. It is unclear if the difficulty with this element of the examination was related to the ability of the examiners to identify systole sonographically, failure to adequately teach this element of the examination or mere oversight of this step. The diameter of the common carotid artery changes roughly 0.51mm (95% CI [0.48–0.54mm]) with flow and this is referred to as flow-mediated vasodilatation, which is dependent on several physiologic factors including blood pressure, arterial compliance, intima-media thickness, smooth muscle tone and neural control mechanisms.^17^,^18^ If the area being measured changes, the VTi also changes. Our study participants frequently failed to measure VTi in systole; however, it is unknown if this error of omission would significantly change the calculation of common carotid flow in a clinically meaningful way.

Our study physicians were taught an entirely new technique using only a brief educational video, and they preferred this learning format. As technology is used increasingly in undergraduate and medical education, residents themselves are accustomed and comfortable with electronic learning formats, perhaps more so than traditional lecture formats.

## LIMITATIONS

This study is limited by a small sample size. Additionally, this study represents findings from one affiliated group of residents and may not be generalizable across other institutions. The physicians in this study have demonstrated the ability to learn the procedure with the reported proficiency levels after a brief instructional video, but it is unknown whether this degree of competency in learning this technique is adequate when determining volume responsiveness in critically ill patients or what the learning curve is to full mastery of this technique. Further study is required to assess the ability of emergency physicians to master the procedure with further practice and instruction. True proficiency in performing the examination for carotid flow measurements would require successful performance of all quality measures consistently with each repetition of the examination. A prospective study evaluating the accuracy of these measurements in a clinical setting would be necessary to truly assess the ability of EPs to accurately and adeptly acquire common carotid flow measurements. The actual utility of this technique in assessing volume status or volume responsiveness in ED patients is unknown. While we found minimal differences between novice and experienced sonographers for the outcome measures, the study was not powered for such an analysis and this is a post-hoc stratification.

## CONCLUSION

Emergency medicine residents at our institution learned a technique for obtaining common carotid artery Doppler flow measurements after viewing a brief instructional video. When assessed at performing this examination, they completed several performance measures with greater than 75% success. There were no differences in ability to perform key elements of the examination between novice and experienced groups. This technique shows promise as a rapid, easy-to-use method of assessing volume status and volume responsiveness.

## Figures and Tables

**Video 1:** Ultrasound of common carotid diameter measurement obtained within 0.5cm of the carotid bulb and measured intima to intima.

**Video 2:** Ultrasound of common carotid Doppler waveform and velocity time integral measurement acquired with an insonation angle of 58 degrees.
